# Mono-Segment Fixation Versus Short-Segment Fixation in Adult Patients With Thoracolumbar Spine Fractures: A Randomized Controlled Trial

**DOI:** 10.7759/cureus.90939

**Published:** 2025-08-25

**Authors:** Vishnu Kumar, Pasupathy Palaniappan, Deep Sharma, Prabu Mounisamy, Gipson T Samuel, Sharran M

**Affiliations:** 1 Orthopedics, Jawaharlal Institute of Postgraduate Medical Education and Research, Puducherry, IND

**Keywords:** implant failure, local kyphosis, mono-segment fixation, perioperative blood loss, sagittal index, short-segment fixation, spine fractures, thoracolumbar fractures

## Abstract

Background

Thoracolumbar spine fractures account for the majority of all spinal injuries and pose a significant challenge in trauma care. Fixation techniques have evolved over the decades, with pedicle screw fixation becoming the norm. However, the debate continues regarding the number of vertebral levels that should be included in the fixation construct for optimal outcomes in case of single-level injuries. While long-segment fixation provides robust stability, it sacrifices motion segments, potentially increasing stiffness and adjacent segment degeneration. Short-segment fixation (SSF), involving one vertebra above and below the fracture, reduces stiffness and implant cost. Mono-segmental fixation (MSF), a recently introduced concept, advocates instrumenting only the fractured vertebra and one adjacent vertebra either above or below. We believe that MSF could provide outcomes similar to SSF with fewer complications, shorter operative times, and reduced blood loss in specific fracture patterns. Hence, this study was designed as a single-center randomized controlled trial to test this hypothesis.

Methodology

Twenty-six patients were randomized into the two intervention groups: Group A (MSF, *n *= 13, 50%) and Group B (SSF, *n *= 13, 50%). After the intervention, the patients followed a standard postoperative care regimen and rehabilitation protocol. All patients underwent open surgery. Radiographs were obtained at regular intervals, and the outcome variables were recorded up to one year following surgery. Sagittal index (SI), anterior vertebral height loss (AVHL), Cobb's angle (CA), local kyphotic angle (LKA), and implant failure were the radiological outcome measures studied. The other parameters assessed were surgical blood loss, postoperative drain volume, surgical time, pain, and wound complications.

Results

The groups were comparable for mean age, gender distribution, AO fracture type, and mechanism of injury (*P *> 0.05). The mean change between observations at one year and immediate postoperative radiographs in SI, AVHL, and CA were similar between the groups (*P* > 0.05). The mean change in LKA was higher in the MSF group (*P* = 0.018, 95% confidence interval (CI) 0.78, 7.70). While the MSF group demonstrated reduced intraoperative blood loss (*P *= 0.055, 95% CI -64.89, -2.80) and surgical time (*P* = 0.039, 95% CI, -24.74, -0.64), both groups had similar drain volume (*P* = 0.443, 95% CI -41.24, -0.21) and pain Visual Analog Scale (VAS) scores throughout the follow-up period (*P *> 0.05).

Conclusions

MSF could be an effective alternative to SSF in selected thoracolumbar fracture patterns with both pedicles and one end plate intact, offering similar radiological outcomes with reduced surgical morbidity.

## Introduction

Thoracolumbar vertebral fractures are one of the commonest spinal injuries [1], the common causes being falls from significant height, motor vehicle collisions, and direct trauma. Vertebral fractures may lead to neural compression due to the retropulsion of bone fragments or by the transfer of the force of injury onto the spinal cord. Backache, instability, segmental kyphosis, and paraplegia are some of the presentations in patients who sustain thoracolumbar vertebral fractures [2]. While bracing and physical therapy are the preferred treatment for stable injuries without neurological involvement, early surgery is necessary when instability or neurological injury is present. Surgical options include posterior decompression and pedicle screw fixation, anterior decompression and fixation and combined anterior-posterior fixation [3]. In recent years, posterior-only decompression and fixation have become the preferred method for addressing most fracture patterns due to advancements in pedicle screw instruments [4]. Long segment fixation was the preferred technique many years back, where two or more levels above and below the fractured vertebra were included in the fixation construct [5]. This technique provided excellent stability to the fracture but resulted in stiffness, adjacent segment stress and extensive surgery while also increasing the treatment cost [6]. Later, the technique of short-segment fixation (SSF) came into practice, where the fractured vertebra is spanned by only one level above and below, sometimes incorporating a screw into the fractured vertebra too. SSF reduces the overall volume and minimizes the additional load on adjacent discs [7]. The optimal number of levels to include in the fixation construct is still debated. According to McCormack's Load Sharing Classification, the decision between long and SSF should be guided by the specific fracture characteristics to achieve optimal treatment results [5]. The main goal of intervention by surgery for thoraco-lumbar vertebral fractures is to decompress the spinal canal, correct any deformities, and stabilize the vertebral column adequately so that early mobilization may be initiated [8]. While achieving that, it is desirable to involve the least number of vertebrae in the fixation construct. This would lead to the preservation of motion segments and a reduction in instrumented levels, thereby reducing blood loss, operative time, wound complications, and possibly deep infections. Hence, mono-segment fixation (MSF) constructs, which included the fractured vertebra along with one adjacent vertebra, either proximal or distal, should be better than SSF if they can provide similar rigidity and pull-out strength and have similar failure rates [8]. Mono-segment pedicle screw fixation has been shown to result in better clinical results accompanied by an insignificant rate of kyphosis recurrence and other complications [9]. Keeping in mind the goals to preserve the motion at more vertebral segments, mono-segment pedicle screw fixation appears to offer a good balance between the extent of fixation and optimum outcome [10]. Unlike short-segment pedicle screw fixation, which spans multiple spinal levels, MSF involves a single segment, thereby reducing the stress placed on adjacent vertebrae and intervertebral discs [11]. Biomechanical evaluations suggest that mono-segment pedicle screw fixation provides rigidity and pull-out strength comparable to short-segment fixation. As a result, it has been used in various AO classification types, including A1, A3, B1, B2, B3, and C fractures [12]. Despite several studies reporting favorable outcomes with MSF, the lack of randomization and control in these studies limits their ability to rigorously evaluate its efficacy and shortcomings with short-segment pedicle screw fixation [13]. To address this research gap, we designed a randomized controlled trial (RCT) to assess the efficacy of mono-segmental pedicle screw fixation compared to short-segment pedicle screw fixation in the treatment of thoracolumbar vertebral fractures.

## Materials and methods

Study design

To assess the radiological outcomes and clinical outcomes of MSF, a single-center, open-label, parallel-group, equivalence RCT was conducted at our institution from July 2022 to December 2024. The trial was registered with the Clinical Trials Registry - India, bearing registration no. CTRI/2022/08/045054 and was approved by the Institutional Ethics Committee for interventional studies (Approval letter no. JIP/IEC/2022/063). A total of 246 patients with thoracolumbar spine fractures were screened, and after obtaining informed consent, 26 eligible participants were randomized into group A (MSF) and group B (SSF) using block randomization with random block numbers to ensure equal allocation in each group. Allocation concealment was ensured using serially numbered opaque sealed envelopes. Since the method of intervention was apparent in the postoperative radiographs, blinding was not possible in this study.

Patients aged more than 18 years with acute thoracolumbar fractures (AO spine classification types A3, B, and C) with vertebral body height loss of more than 30% or with neurological deficits were included in the study. Inclusion also demanded that one end plate and both pedicles of the fractured vertebra should be intact to allow for MSF. Multi-level fractures requiring fixation and pathological fractures were excluded.

Intervention

**Figure 1:**
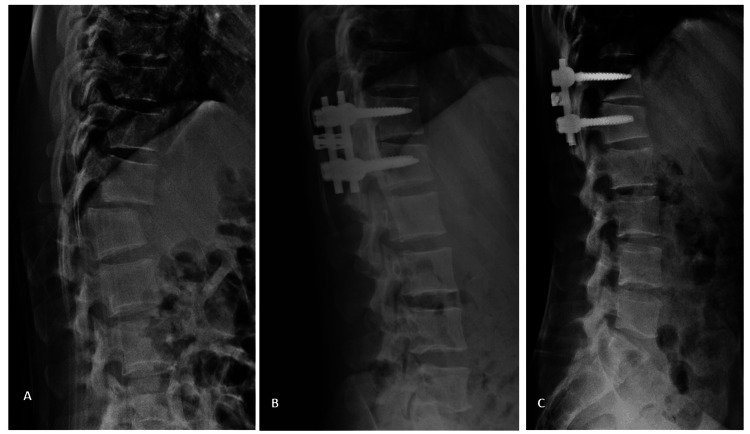
A 32-year-old male with AO type C D12–L1 injury. (A) Preoperative radiograph showing the dislocation. (B) Postoperative radiograph following reduction and mono-segment fixation. (C) Postoperative radiograph at one year demonstrating intact fixation.

In Group A, MSF was performed [13] with pedicle screws inserted into the fractured vertebra and adjacent vertebra, either proximal or distal, depending on which end plate of the fractured vertebra is disrupted (Figure 1).

**Figure 2:**
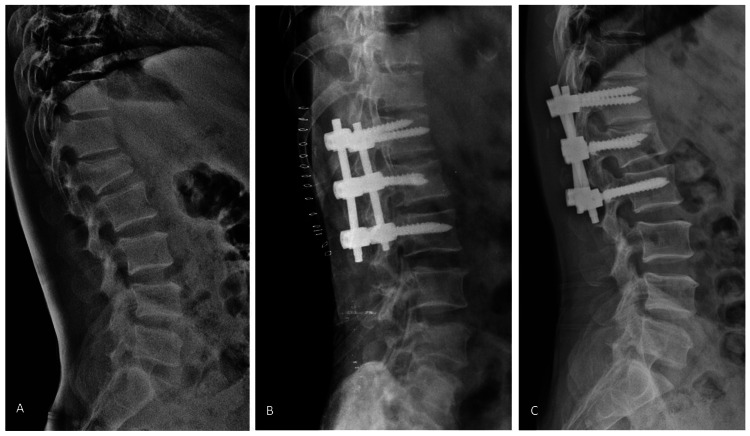
A 45-year-old male with AO type B2 L1 injury. (A) Preoperative radiograph. (B) Postoperative radiograph after short-segment fixation. (C) Radiographs at one year showing healing and intact fixation.

In Group B, SSF [14] was performed with pedicle screws placed in the fractured vertebra and the adjacent vertebra (Figure 2).

Pedicle screws were directed away from the location of the fracture in the fractured vertebrae. All patients were treated with open surgery using a standard posterior midline approach. In patients with canal occlusion greater than 50% with neurological deficits, a laminectomy was done for direct decompression while preserving the spinous process and posterior ligamentous complex (PLC). Pedicle screws of diameter 6.5mm and maximum possible length (40-45 mm) were used in all the patients, along with transverse connectors in MSF to ensure optimal pull-out strength. However, we did not attempt spinal fusion in any of the patients. All patients followed a standard rehabilitation protocol, and a spinal brace was used in the initial postoperative period in patients with excessive pain.

Outcome measures

Primary outcomes included radiological parameters such as sagittal index (SI), anterior vertebral height loss (AVHL), Cobb's angle (CA), and local kyphotic angle (LKA), measured at regular intervals up to one year after surgery [13,15,16]. Secondary outcome measures were intraoperative blood loss, postoperative drain output, duration of surgery, wound complications, implant failure, and pain measured using a 10-point Visual Analog Scale (VAS) [17] at various time points. The outcomes were assessed by a single assessor.

Sample size

The sample size was calculated as 13 in each group, anticipating a true mean difference and standard deviation in the SI between MSF and SSF groups of 2.3 degrees and 2.5 degrees [13], with an equivalence margin of 4 degrees, and a one-sided level of significance of 5% and power of 80% power.

Statistical analysis

**Figure 3:**
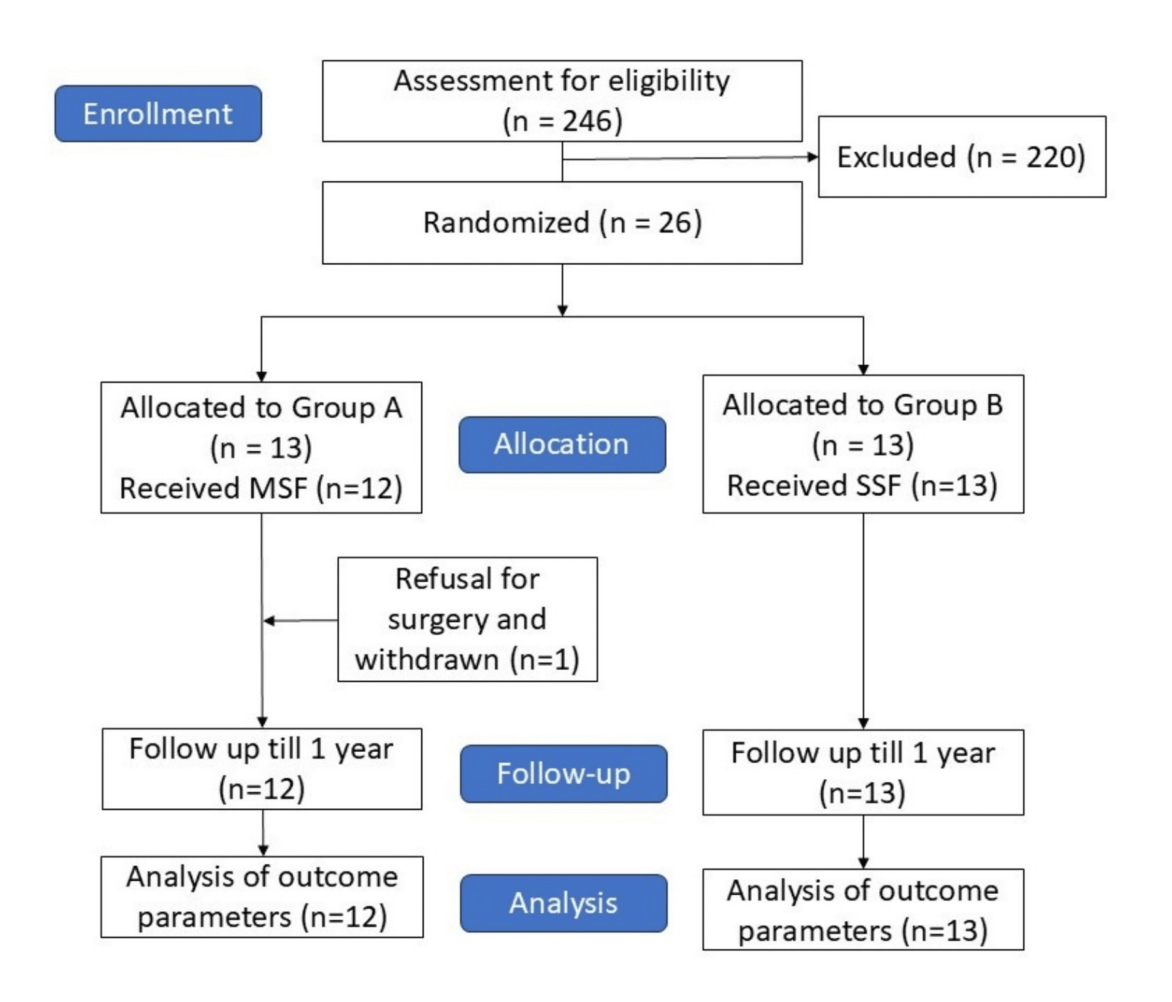
CONSORT diagram depicting the flow of participants in the trial. CONSORT, Consolidated Standards of Reporting Trials; SSF, short-segment fixation

Intention-to-treat analysis was done using IBM SPSS Statistics software version 19 (IBM Inc., Armonk, NY). All statistical tests were carried out at 5% level of significance. The participant flow is depicted in the Consolidated Standards of Reporting Trials (CONSORT) diagram (Figure 3).

## Results

One patient from the MSF group decided not to undergo surgery after randomization and hence was withdrawn from the trial. All patients were closely followed for one year following surgery. The population characteristics of both groups are summarized in Table 1. Both groups were comparable in mean age, gender distribution, mechanism of injury, AO fracture type, and time to surgery (*P *> 0.05).

**Table 1:** Comparison of basic traits of the study groups. Age and time to surgery are expressed as mean (SD) and compared using the Independent t-test. Gender, injury mechanism, and fracture classification are expressed as frequency (percentage) and compared using the chi-square test. SD, standard deviation; SSF, short-segment fixation; MSF, mono-segment fixation

Parameter	Group MSF (*n* = 12)	Group SSF (*n* = 13)	Test statistic (*t*/*X*^2^)	*P*-value
Age (years)	40 (11.92)	37.85 (12.41)	0.451	0.656
Gender, *n* (%)				
Male	10 (83.3%)	9 (69.2%)	0.645	0.317
Female	2 (16.7%)	4 (30.9%)		
Injury mechanism, *n* (%)				
Fall from height	6 (50%)	7 (53.9%)	0.037	0.848
Road traffic accident	6 (50%)	6 (46.1%)		
AO spine fracture classification, *n* (%)				
A3	6 (50%)	6 (50%)	0.828	0.843
B1	1 (8.3%)	1 (7.7%)		
B2	2 (16.7%)	4 (30.8%)		
C	3 (25%)	2 (15.4%)		
Time to surgery (days)	6 (2.54)	5.53 (2.87)	0.433	0.669

Radiological outcomes

Changes in the radiological outcome measures between immediate postoperative radiographs and radiographs at one year are summarized in Table 2. Both MSF and SSF groups demonstrated significant improvement in SI, AVHL, and CA after surgery. However, there was no difference between the groups at one year (*P *> 0.05). The change in LKA with time was found to be lower in the SSF group at one year (*P *= 0.0184, 95% CI 0.78, 7.70).

**Table 2:** Comparison of radiological outcome measures. The figures represent the mean change in the parameters measured at the immediate postoperative period and at one year. SD, standard deviation; SSF, short-segment fixation; MSF, mono-segment fixation

Parameter	Group MSF (Mean, SD) (*n* = 12)	Group SSF (Mean, SD) (*n* = 13)	Test statistic (*t*)	95% confidence interval (CI)	*P*-value
Sagittal index (degrees)	8.31 (3.83)	5.61 (5.28)	1.48	-1.05, 6.43	0.150
Anterior vertebral height loss (mm)	13.88 (8.33)	8 (8.26)	1.8	-0.84, 12.60	0.083
Cobb angle (degrees)	8.69 (4.98)	6.47 (4.15)	1.08	-1.99, 6.43	0.289
Local kyphosis angle (degrees)	9.94 (3.99)	5.7 (4.53)	2.52	0.78, 7.70	0.018

Clinical outcomes

Mean preoperative pain VAS scores were higher in the MSF group (*P* = 0.018, 95% CI 1.89, 7.40). The scores reduced to <1 at 12 months in both groups, with no difference between them (*P *= 0.612) at one year (Table 3).

**Table 3:** Comparison of pain VAS scores. VAS, Visual Analog Scale; SSF, short-segment fixation; MSF, mono-segment fixation

Time point	Group MSF (Mean, SD) (*n* = 12)	Group SSF (Mean, SD) (*n* = 13)	Test statistic (*t*)	95% confidence interval (CI)	*P*-value
Preoperative	9.94 (3.99)	5.7 (4.53)	2.52	1.89, 7.40	0.018
Postoperative	5.15 (1.9)	4.53 (0.77)	1.07	-0.56, 1.80	0.292
6 weeks	2.23 (1.16)	2.38 (1.04)	0.35	-1.05, 0.74	0.726
3 months	1.46 (1.05)	1.07 (1.11)	0.9	-0.49, 1.26	0.374
6 months	0.46 (0.24)	0.3 (0.17)	0.51	-0.46, 0.77	0.612
12 months	0.32 (0.16)	0.28 (0.12)	0.67	-0.23, 0.69	0.741

The MSF group had lower surgical blood loss (*P* = 0.055; 95% CI -64.89, -2.80) and shorter surgical time (*P* = 0.039; 95% CI -24.74, -0.64), with similar postoperative drain output (*P* = 0.443; 95% CI -41.24, -0.21), compared with the SSF group (Table 4). 

**Table 4:** Comparison of surgical parameters. Intraoperative blood loss and postoperative drain volume are expressed as median (IQR) and compared using the Mann-Whitney U test. Surgical time is expressed as mean (SD) and compared using the Independent t-test. IQR, interquartile range; SD, standard deviation; SSF, short-segment fixation; MSF, mono-segment fixation

Parameter	Group MSF (*n* = 12)	Group SSF (*n* = 13)	Test statistic (*U*/*t*)	95% confidence interval (CI)	*P*-value
Intraoperative blood loss (mL)	150 (60)	190 (10)	35.5	-64.89, -2.80	0.055
Postoperative drain volume (mL)	20 (45)	40 (60)	57.5	-41.24, 2.21	0.443
Surgical time (minutes)	99.23 (17.54)	111.92 (11.64)	2.17	-24.74, -0.64	0.039

Complications

No patients had implant-related complications in the groups. One patient in the SSF group had superficial wound dehiscence, which was managed with dressings. None of the patients required revision surgery.

## Discussion

In our exploratory trial, the mean change in the radiological outcome measures, especially SI, AHL, and CA, was comparable between the groups. The SI had good correction observed in the immediate postoperative radiographs in both groups, which was maintained or even increased at the final follow-up, suggesting maintenance of fracture reduction and accommodation by the adjacent discs to the initial restoration of the vertebral height. Although the mean SI at one year was higher in the SSF group, it was not significant (Table 2), highlighting the ability of MSF to hold the fracture in reduction and allow for progressive healing. Similar to SI, the AVHL percentage also showed no meaningful difference between the groups, although the MSF group recorded an increase with time (5.88%). This finding further demonstrates that MSF was comparable to SSF in maintaining vertebral height after fixation. The change in mean CA over time also revealed only a minimal difference between groups; mean values in the MSF group were higher by 2.21 degrees, a difference that was not clinically significant. Similar results were reported by Wei et al. in their RCT evaluating MSF in patients with AO type A3 fractures without weakness [13]. Similar results were also reported in a retrospective comparison of 48 patients with a follow-up period of 30 months. The authors found MSF to be as effective as SFF in restoring vertebral height and correcting kyphosis, especially in well-selected AO type A and B fractures [18]. They had also evaluated the vertebral compression percentage in a separate retrospective cohort with 20 patients who had undergone MSF and reported that most of the restored vertebral compression percentage was unchanged at the final follow-up [19]. This also demonstrated the effectiveness of MSF in certain fracture types, although spinal fusion was performed in all their patients. Ibrahim et al. reported excellent maintenance of radiological parameters following MSF in their prospective cohort of 40 patients. Their intervention also included anterior reconstruction with a transforaminal lumbar interbody fusion (TLIF) cage in a few patients [20].

We found a significant increase of 5.70 degrees in LKA progression in the MSF group compared with the SSF group (*P* = 0.018; 95% CI 0.78, 7.70). This finding was contrary to the observations of La Maida et al. [19] and Bhattarai and Shah [10], who had reported little increase in the kyphosis angle following MSF. However, this difference could be because of a multitude of factors like study design, fracture characteristics, demographic profile of the study population, etc. While the other radiological parameters were similar between the groups in our study, the significant difference in LKA could be due to the absence of the accommodative effect of adjacent disc spaces. A suitable sample size might reveal the actual effect of MSF on LKA.

MSF involves fixation of fewer levels when compared to SSF, and hence it’s only logical that the operation time for MSF is lower. Also, MSF involves shorter surgical exposure since fewer vertebrae are instrumented. Accordingly, we observed that the surgical time and intraoperative blood loss were significantly lower in the MSF group, although the difference in the postoperative drain output was not significant (Table 3). This was also reported by Bhattarai and Shah [10], who found lower operating time in MSF and similar surgical blood loss between MSF and SSF in patients with AO type A3 fractures. We found that both techniques were effective in controlling pain, and there was a significant and persistent reduction in pain VAS scores over time. We had used spinal bracing postoperatively till suture removal in a few patients in both groups to aid in rehabilitation. While MSF resulted in greater initial pain relief, both groups reported similar VAS scores of 1 at the final follow-up (Table 4).

None of the patients had implant failure or needed revision surgery. Interestingly, our study also included three patients in the MSF group with AO Type C fractures, one each at D10-D11, D11-D12, and D12-L1. These patients, too, had no implant-associated complications and progressed to heal well. This adds to the fact that MSF has adequate biomechanical properties to treat AO type C fractures.

Owing to the nature of the study design, bias was prevented to a reasonable extent. The outcomes were assessed by the same assessor who was not a part of the surgical team, thereby minimizing observer and inter-observer bias. Despite being a well-designed RCT, our study had a few limitations. The sample size was calculated based on an equivalence margin of 4 degrees, which was based on expert opinion, as the literature was lacking. Although 246 patients were screened, only 26 satisfied the inclusion criteria. The small sample size prevented us from evaluating the efficacy of MSF in specific fracture types. The follow-up period of one year in this study might be adequate to assess fracture union and spinal alignment, but it is very short to assess adjacent segment disease, which is considered to be one of the advantages of MSF. Cost-effective analysis was not a part of our trial. If included, it could have provided valuable insights into the economics of spine fracture treatment.

## Conclusions

MSF could be an effective alternative to SSF in certain fracture types where the fractured vertebra has at least one end plate and both pedicles intact. Accordingly, AO type A1, A3, B, and C fractures are amenable to treatment with MSF. In our study, MSF has demonstrated radiological outcomes and pain control similar to SSF, while resulting in shorter surgical time. Large, multicenter trials are needed to establish long-term outcomes and recommend general adoption of the technique.
